# Spontaneous lesser omental herniation resolved by laparoscopic surgery: case report and systematic literature review

**DOI:** 10.1007/s00464-023-10279-4

**Published:** 2023-07-21

**Authors:** André S. Alves, Alexandre Balaphas, Katie Zuo, Philipp Hauser, Angeliki Neroladaki, Toni Raffoul

**Affiliations:** 1 Faculty of Medicine, University of Geneva, 1211 Geneva, Switzerland; 2 Department of Surgery, Neuchâtel Hospital Network, Maladière 45, 2000 Neuchâtel, Switzerland; 3 Faculty of Life Sciences & Medicine, King’s College London, London, SE1 1UL UK; 4 Division of Radiology, Hôpital de la Tour, 1217 Geneva, Switzerland; 5 Division of Digestive Surgery, Hôpital de la Tour, 1217 Geneva, Switzerland

**Keywords:** Lesser omentum, Small omentum, Gastrohepatic omentum, Hernia, Intra-abdominal

## Abstract

**Background:**

Despite its extremely low incidence, intra-abdominal herniation through the lesser omentum is associated with a high mortality rate and must be recognized early and treated urgently. To overcome a lack of data on the management of this condition, we collected and reviewed all the reported cases of operated lesser omental hernia and presented the case of a patient treated by laparoscopy for an isolated lesser omental hernia.

**Methods:**

According to PRISMA guidelines and using PubMed, Cochrane Library, and Web of Science, a systematic literature review of cases of lesser omental hernia treated by surgery was performed on February 12, 2023.

**Results:**

Of 482 articles, 30 were included for analysis and only 9 articles presented an isolated hernia through the lesser omentum. Among these, 4 patients were female and the median age was 38. Upper abdominal pain and vomiting were reported in 7 out of 9 patients. The small bowel was responsible for 78% (7/9) of all lesser omental herniations. All of them were treated by laparotomy. In addition, we describe the case of a 65-year-old woman without prior surgical history who was treated by laparoscopy for a spontaneous closed loop hernia through the lesser omentum without any other associated hernias.

**Conclusion:**

Mostly associated with prior surgery or trauma, this type of herniation could sometimes occur spontaneously without any sign of peritonitis. Due to the high mortality rate, internal abdominal hernias should always be ruled out with a CT scan in front of patients presenting with persisting acute abdominal pain and no alternative diagnosis.

**Supplementary Information:**

The online version contains supplementary material available at 10.1007/s00464-023-10279-4.

Transomental herniations are rare conditions, mostly reported in patients over the age of 50 years old [1]. An abnormal omental opening can be either acquired following abdominal surgery, trauma, inflammatory conditions, low body mass index (BMI) or be due to congenital defects and be associated with a long mesentery, intestinal malrotation, or abnormal peritoneal attachments [2–6]. Although internal hernias are extremely rare and represent between 1 and 4% of acute or intermittent intestinal obstructions, it is essential to not miss this diagnosis [7, 8]. Emergency management of omental hernias by surgery is critical as postoperative mortality rate is over 30% and even 50% if strangulation is present [3, 9]. The lack of current literature on this rare condition, particularly for lesser omental hernias, which can present with nonspecific signs and symptoms, makes diagnosis and management difficult.

A classification of lesser omental hernia has been proposed by Kitagishi et al. [10]. Two forms of herniation were described, where type 1 is defined as a prolapse through the lesser omentum into the lesser sac (abdominal cavity → omental bursa) while type 2 is characterized by a prolapse from the retrogastric space into the abdominal cavity (foramen of winslow or free retrogastric space following colectomy → omental bursa → abdominal cavity). Chen et al. suggested another classification to specify herniation pathway and its relation with omental bursa [11]. In this classification, type 1 defines herniation through the greater omentum, type 2 through the foramen of Winslow, type 3 through the transverse mesocolon, and type 4 directly through the lesser omentum only, i.e., without associated hernias (Fig. 1; Supplementary Table 1).

**Fig. 1:**
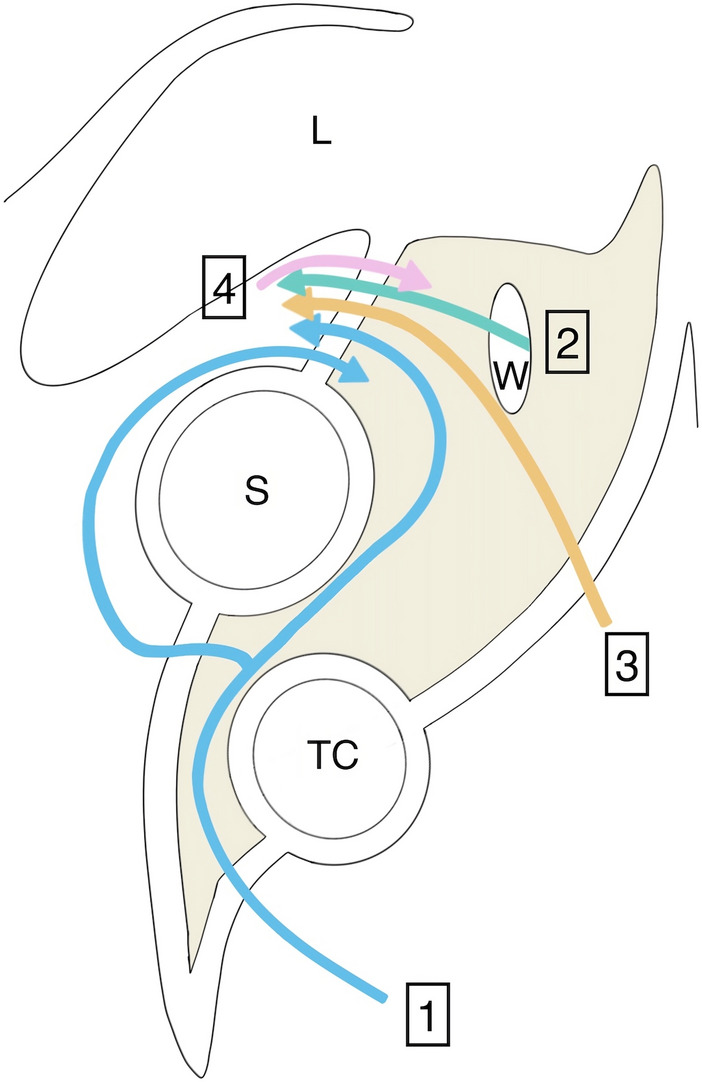
Lesser Omental Hernias Classification according to Chen et al. *L* liver, *S* stomach, *TC* transverse colon, *W* foramen of Winslow, *1* Hernia through the greater omentum and through the lesser omentum, *2* Hernia through the foramen of Winslow and lesser omentum, *3* Hernia through the transverse mesocolon and the lesser omentum, *4* Hernia through the lesser omentum only

To add to the scientific knowledge of this rare manifestation in accordance with the guidelines for surgical case reports (SCARE) [12], we report the case of a patient with isolated intestinal herniation through the small omentum only and performed a PRISMA systematic literature review on this topic.

## Methods

Regarding the case presentation, data was retrieved from patient records and presented according to the SCARE 2020 guidelines. Guidelines do not require IRB approval.

We then performed a systematic literature review in accordance with the PRISMA 2020 guidelines [13]. This systematic review was conducted on February 12, 2023 using the following databases: PubMed, Cochrane Library, and Web of Science. The artificial intelligence literature mapping tool ResearchRabbit (Human Intelligence Technologies Incorporated, Seattle, WA, USA, https://www.researchrabbit.ai) was used to collect additional articles. Language was restricted to English. The search terms used in the title, abstract, Medical Subject Headings, and keyword fields included combinations as follows: (((lesser omentum) OR (small omentum)) OR (gastrohepatic omentum)) AND (hernia).

All articles presenting in English with a case of lesser omental herniation were selected for analysis. No limitations were applied on the age of the patients or their ethnicity. Conference abstracts, simulation studies and clinical studies in non-human subjects, unpublished studies, and other studies in a foreign language were not included. Studies involving patients who presented other types of intra-abdominal herniations were also removed.

Two authors (ASA and AB) independently identified the relevant studies based on the title and the abstract. Selected articles were then fully read. If the studies met all selection criteria, data were extracted independently by the two authors. Disagreement was resolved after consultation with the senior author (TR).

The following variables were extracted: the name of the first author, the publication year, the patient age and gender, types of symptoms, the associated hernias, the herniated organ, surgical history, abdominal trauma history, and the type of surgical treatment. Two authors (ASA and AB) independently identified these parameters.

Through collecting all the current evidence of this condition, we hope to gain better insight into this rare phenomenon.

## Case presentation

A 65-year-old woman was admitted to the emergency department for an upper abdominal pain scaled at 10/10 in the visual analogue scale (VAS) [14, 15]. The pain was associated with nausea and alimentary vomiting but without transit disorder, dyspnea, or chest pain. She presented for the first time these symptoms. Patient’s history was negative for any systemic diseases, prior abdominal surgeries or trauma and her BMI was of 26. At admission, vital signs were all within normal ranges. Bowel sounds were audible and of normal tonality. Clinical examination only revealed epigastric and right upper quadrant tenderness but a negative Murphy’s sign. Laboratory tests were normal except for a mildly elevated leukocytosis 10.8 G/l. A point-of-care ultrasound highlighted a gallstone in the gallbladder. She received Tramadol which reduced the pain to 3/10. The diagnosis of biliary colic was retained and the patient was discharged with a 24-h follow-up appointment.

**Fig. 2:**
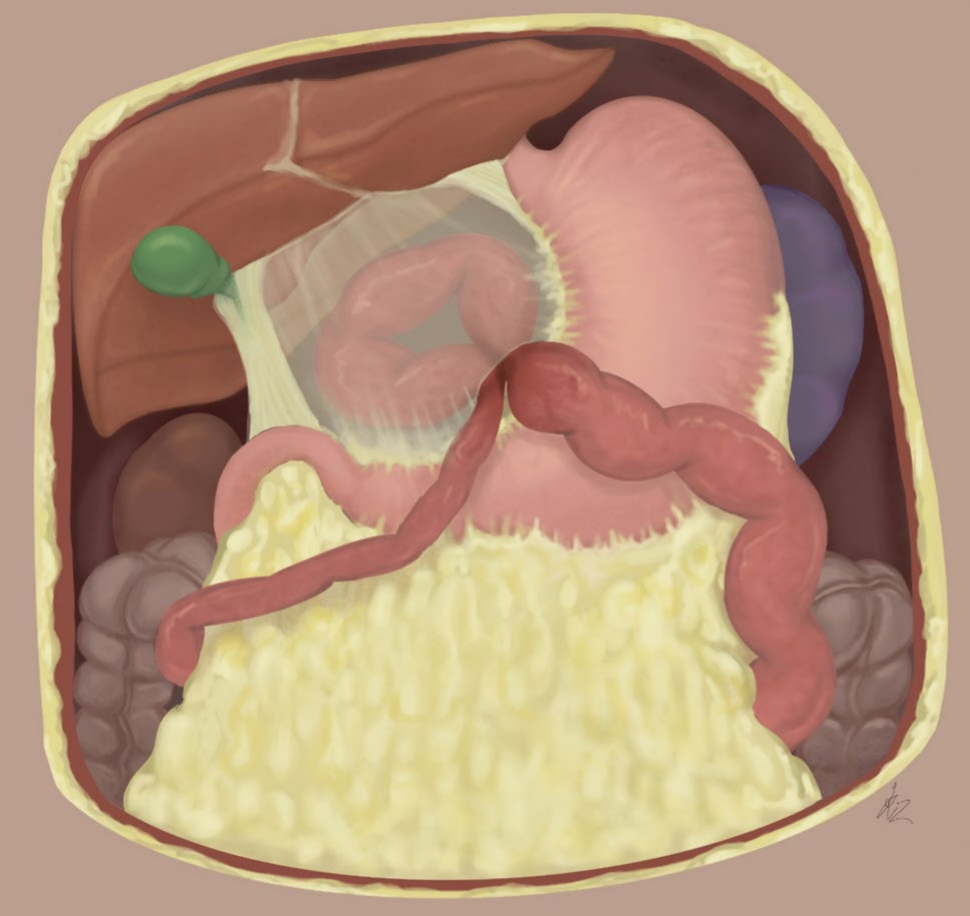
65-Year-old female with isolated spontaneous lesser omental hernia

**Fig. 3:**
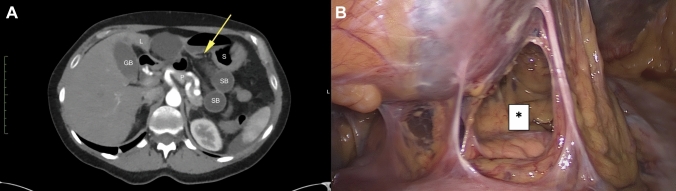
**A** Abnormally positioned dilated small-bowel (SB) internal hernia between the liver (L), the stomach (S), the pancreas (P) and the gastro-hepatic ligament (yellow arrow) signing closed loop ileus in the lesser omental sac. **B** Lesser omentum after resolving the herniation by laparoscopy; *Lesser omentum defect (Color figure online)

At follow-up appointment, the patient described persistent mild pain in the upper abdominal region. Laboratory tests were normal except for a mildly elevated C-reactive protein. A complete US did not find any signs of acute cholecystitis, but a moderate quantity of free intraperitoneal fluid was detected. A total abdominal computed tomography (CT) scan showed a “closed loop” small-bowel internal hernia of the lesser omentum and free intraperitoneal fluid along the dilated upward ileal loops, in the pouch of Douglas and in peri-splenic localisation (Figs. 2, 3).

The patient was admitted to the operation room for emergency laparoscopy surgery (Fig. 3). Laparoscopy confirmed the presence of a small-bowel loop incarcerated into the omental bursa through a gap into the lesser omentum. The small bowel was reduced carefully with a delicate traction avoiding vessel damage. The herniated intestinal segment was hyperemic with meso-infiltration but quite viable and it was decided to not proceed to resection. The lesser omental defect was enlarged and the adherences were dissected. Due to concerns regarding concomitant biliary colic, cholecystectomy was also performed. The entire operation lasted 61 min. No postoperative complications occurred, and the patient was discharged on postoperative day 4. Postoperative follow-up was completely normal at 1 week and at the various follow-up examinations. The patient’s pain disappeared, and she regained full function.

## Results

**Fig. 4:**
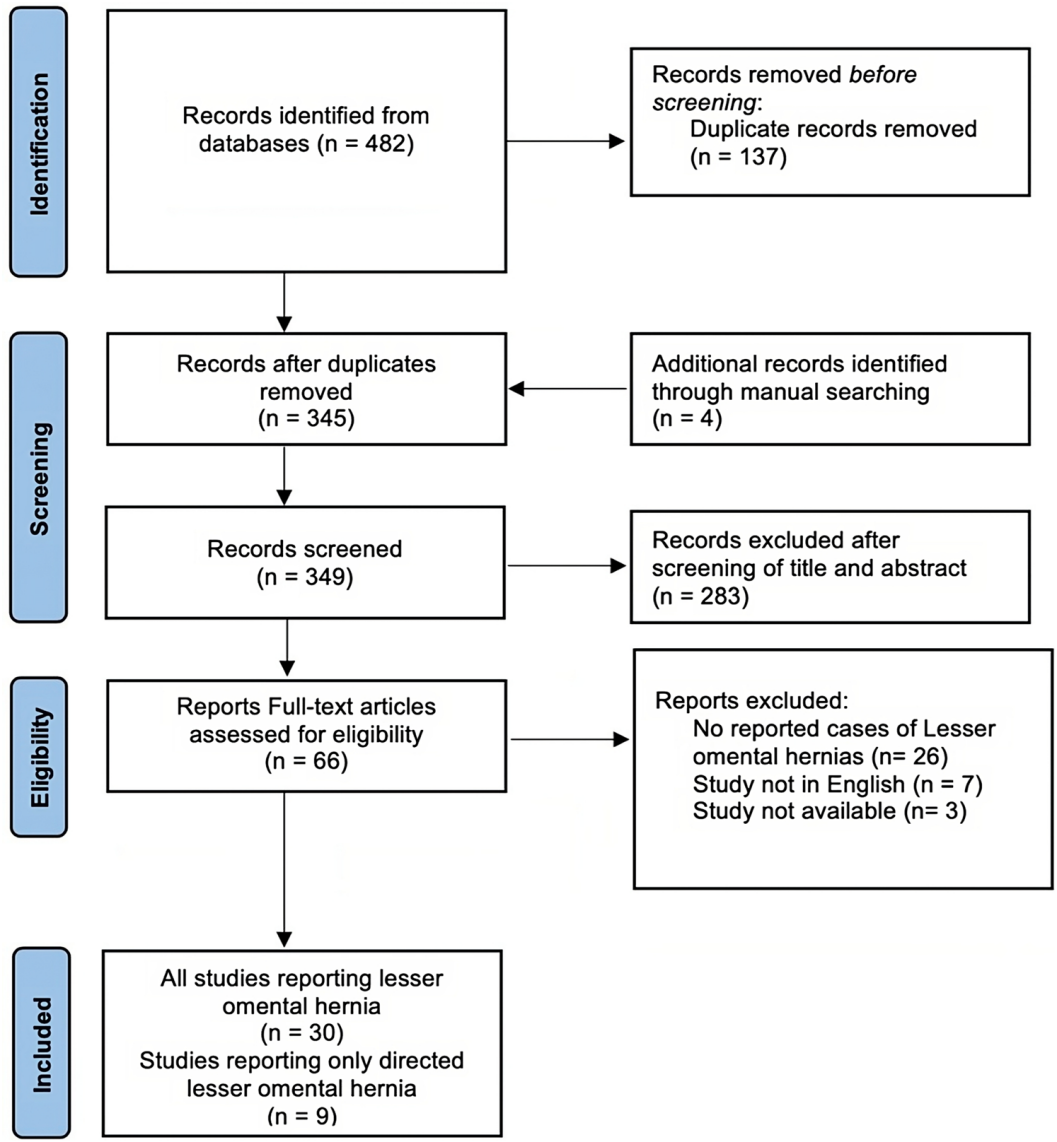
Flow diagram of searching eligible studies

Among 482 studies, only 30 available articles of which were case reports, described patients with a lesser omentum hernia in English (Fig. 4). In total, thirty patients were reported including 15 females (Table 1). The median age at diagnosis was 43.5 (14–88). A CT scan was performed preoperatively in 63% (19/30) of cases. The small bowel was responsible of 87% (26/30) of all lesser omental hernias. Among lesser omental hernias, 33% (10/30) were associated with a gastrocolic ligament hernia first, 13% (4/30) with a mesocolon hernia first and 10% (3/30) with a foramen of Winslow hernia first. Prior abdominal surgery was present among 23% (7/30) of patients where 5 of them underwent partial or total colectomy. Emergency laparotomy was performed in 87% (26/30) of the case, while 14% (4/30) were managed with laparoscopy.

**Table 1:** Selected articles reporting lesser omental hernia

Studies	Age	Gender	Symptom	CT	Chen vs Kitagishi classification	Associated hernia	Herniated organ	Previous surgery or trauma	Surgery
Aylett, 1946 [16]	34	M	UAP	No	4/1	None	Small bowel	None	Laparotomy
Baek, 1994 [17]	88	F	UAP, vomiting	Yes	4/1	None	ileum	None	Laparotomy
Bahadori, 2003 [29]	14	F	AP	No	3/2	Mesocolon	Small bowel	Appendectomy	Laparotomy
Chattapadhyay, 1982 [30]	28	F	AP, vomiting	No	1/2	Gastrocolic lig	Small bowel	None	Laparotomy
Chen, 2012 [11]	56	F	UAP, vomiting	Yes	1/2	Gastrocolic lig	Jejunum	None	Laparotomy
Chou, 2005 [31]	17	F	UAP	Yes	3/2	Mesocolon	Small bowel	NA	Laparotomy
Coulier, 2007 [18]	79	F	UAP, vomiting	Yes	4/1	None	Stomach	Na	Laparotomy
Duarte, 2002 [19]	36	M	AP, vomiting	No	4/1	None	ileum	NA	Laparotomy
Gants, 1953 [20]	30	M	UAP, vomiting	No	4/1	None	Stomach, transverse colon	Abdominal trauma	Laparotomy
Hanatate, 1997 [32]	45	F	UAP, vomiting	No	1/2	Gastrocolic lig	ileum	None	Laparotomy
Inoue, 1996 [33]	49	M	UAP	Yes	1/2	Gastrocolic lig	ileum	None	Laparotomy
Joao, 2022 [21]	44	M	UAP, vomiting	Yes	4/1	None	Jejunum	Open appendectomy	Laparotomy
Jolliffe, 2021 [34]	77	M	AP	Yes	1/2	Gastrocolic lig	Jejunum	Open subtotal colectomy	Laparotomy
Konishi, 2014 [25]	42	M	UAP, vomiting	Yes	-/2	None	Jejunum	Total colectomy	Laparotomy
Kulkarni, 2019 [35]	54	F	NA	Yes	3/2	Mesocolon	Proximal small bowel	Hysterectomy	Laparoscopic
Kundaragi, 2014 [36]	55	M	AP, vomiting	Yes	3/2	Mesocolon	Small bowel	None	Laparotomy
Lee, 2016 [37]	57	M	UAP, vomiting	Yes	1/2	Gastrocolic lig	Small bowel	None	Laparotomy
Li, 2017 [22]	38	F	UAP	No	4/1	Gastrocolic lig	ileum	None	Laparotomy
Liu, 2020 [26]	73	F	AP	Yes	-/2	None	Small bowel	Total colectomy	Laparotomy
Masubuchi, 2012 [27]	57	F	vomiting	Yes	-/2	None	Small bowel	Transverse colectomy	Laparoscopy
Min, 2010 [38]	47	F	UAP	Yes	1/2	Gastrocolic lig	Jejunal	None	Laparotomy
Rathnakar, 2016 [23]	54	M	AP, vomiting	No	4/1	None	Jejunum	None	Laparotomy
Rich, 2002 [39]	67	F	UAP	No	2/2	Winslow	cecum and right colon	None	Laparotomy
Saida, 2000 [40]	33	M	UAP	Yes	2/2	Winslow	ileum	None	Laparotomy
See, 2002 [41]	41	M	UAP, vomiting	No	1/2	Gastrocolic lig	ileum	None	Laparotomy
Talebpour, 2005 [42]	29	M	UAP, vomiting	Yes	1/2	Gastrocolic lig	Jejunum	None	Laparoscopy
Tjandra, 1991 [43]	43	F	UAP	Yes	2/2	Winslow	Caecum, ascending and proximal colon	NA	Laparotomy
Tran, 1991 [24]	24	F	UAP, vomiting	Yes	4/1	None	Jejunum	None	NA
Ugianskis, 2022 [28]	35	M	UAP, vomiting	Yes	-/2	None	Duodenum, uncinated part of the pancreas	Total colectomy	Laparoscopy
Yasuda, 1989 [44]	20	M	UAP, vomiting	No	1/2	Gastrocolic lig	ileum	None	Laparotomy

*UAP* upper abdominal pain, *AP* abdominal pain, *F* female, *M* male

Of these 30 studies, only nine cases presented a direct herniation through the small omentum into the lesser sac as in the presented case and was also defined as type 1 according to Kitagishi classification or type 4 according to Chen classification (Supplementary Table 2) [16–24]. Four studies were not included in these 9 because the patients had no transverse mesocolon or gastrocolic ligament following a previous total or partial colectomy [25–28]. In almost all case except one, there were no prior history of previous abdominal surgeries, with the only exception being an appendectomy [21]. Among the spontaneous direct lesser omental hernias, four patients were female and the median age was 38 (24–88). Upper abdominal pain and vomiting were the main symptoms, reported in 78% of patients (7/9). Diagnosis made by computed tomography scan was done in 44% (4/9) of cases. The small bowel was responsible of 78% (7/9) of all lesser omental herniations. In all these cases laparotomy was performed to relieve the obstruction and to confirm the diagnosis. Thus, to the best of our knowledge, we reported the first case of a spontaneous lesser omental hernia treated by laparoscopic surgery.

## Discussion

We presented the first case of a patient with a lesser omental hernia, treated successfully with laparoscopic surgery alone. A lesser omental hernia diagnosis is challenging as symptoms are variable and can overlap with other abdominal diseases. In this case, the patient had no risk factors for an intra-abdominal hernia. The laboratory tests were reassuring and the diagnosis of a biliary etiology masked the clinical picture of bowel strangulation. While the exact cause of lesser omental defect was not identified, anatomic predisposition was probably responsible of the spontaneous bowel herniation into the omental bursa.

Internal herniation describes the protrusion of a visceral content through a normal or an abnormal aperture within the abdominal cavity [45]. Including the gastrohepatic and hepatoduodenal ligaments, the small omentum is constituted of a double layer of peritoneum. In some cases, this structure has areas of weakness and may allow viscera to pass through. According to our review, the small intestine was found in 87% of herniations through the lesser omentum.

Traditionally, plain abdominal radiography was a basic first-line diagnostic imaging. However, interpretation of a plain abdominal radiograph is challenging and past studies have shown low sensitivity in acute abdominal pain evaluation with this imaging tool [46]. Recent and advanced diagnostic imaging, specifically computed tomography (CT) provides a more accurate diagnosis [47]. CT is an excellent supportive tool to define bowel obstruction and provide diagnostic hallmarks for intra-abdominal hernias [48, 49]. In a direct lesser omental hernia, a CT scan was performed in 4 of 9 studies. In Rathnakar et al. study, the CT was not available and in the Li et al. study the patient was unstable, so laparotomy was performed immediately. The three last studies reported patients before 1996 and plain abdominal radiography was used more often in those times. Nowadays, CT scan should be used as the first-line imaging of choice for non-resolving acute abdominal pain without an alternative diagnosis [50]. If the prognosis is life-threatening, surgery should not be delayed by an imaging test. The final diagnosis will be made during the operation.

In the reviewed cases most surgeries were performed by laparotomy and for direct lesser omental hernias all cases were performed by laparotomy. This could be related to the emergent character of bowel obstruction but also to the anatomic specificity of this region. Indeed, complex cases typically including strangulation require enlargement of the hernia sac dissection which due to the localization might be more confidently performed by laparotomy. However, depending on the situation, a simple traction on the herniated viscera could be enough to remove the obstruction. Only gentle traction must be applied to avoid loops or portal pedicle injuries. However, as presented in our case, laparoscopic management was feasible and prove to be an effective strategy to confirm the diagnosis and reduce the hernia.

The limitations of the study are mainly due to the lack of existing data, mostly explained by how uncommon this type of hernia is. Only the lack of case reports precluded us from performing a meta-analysis. However, we now present for the first time, all the current evidence regarding lesser omental hernias and describe the first laparoscopic case of a spontaneous hernia through the lesser omentum.

## Conclusion

Mild abdominal pain and vomiting could hide an internal abdominal hernia. Past abdominal trauma or surgery are risk factors for lesser omental hernias. A CT scan should be the first-line imaging of choice for persistent acute abdominal pain without alternative diagnosis when the patient is stable. Laparoscopic surgery offers a safe and reliable approach to confirm the diagnosis and reduce lesser omental hernias.

## Supplementary Information

Below is the link to the electronic supplementary material.

Supplementary file1 (DOCX 12 kb)

Supplementary file2 (DOCX 18 kb)

## Data Availability

Not applicable.
