# Supplementary material for: Spontaneous lesser omental herniation resolved by laparoscopic surgery: case report and systematic literature review
Source: Surg Endosc. 2023 Jul 21;37(9):6704–10. doi: 10.1007/s00464-023-10279-4 (PMC10462501; doi:10.1007/s00464-023-10279-4)
Supplement: Supplementary file 1 — Supplementary file1 (DOCX 12 kb) [file 464_2023_10279_MOESM1_ESM.docx]

| **Types** | **Herniation Pathway** |
| --- | --- |
| I | Hernia through the greater omentum and through the lesser omentum |
| II | Hernia through the foramen of Winslow and lesser omentum |
| III | Hernia through the transverse mesocolon and the lesser omentum |
| IV | Hernia through the lesser omentum only |
