# Supplementary material for: Spontaneous lesser omental herniation resolved by laparoscopic surgery: case report and systematic literature review
Source: Surg Endosc. 2023 Jul 21;37(9):6704–10. doi: 10.1007/s00464-023-10279-4 (PMC10462501; doi:10.1007/s00464-023-10279-4)
Supplement: Supplementary file 2 — Supplementary file2 (DOCX 18 kb) [file 464_2023_10279_MOESM2_ESM.docx]

| **Author** | **Age** | **Gender** | **Symptom** | **CT** | **Associated Hernia** | **Herniated Organ** | **Previous Surgery or Trauma** | **Surgery** |
| --- | --- | --- | --- | --- | --- | --- | --- | --- |
| Aylett, 1946 (16) | 34 | M | UAP | No | None | Small bowel | None | Laparotomy |
| Baek, 1994 (17) | 88 | F | UAP, vomiting | Yes | None | ileum | None | Laparotomy |
| Coulier, 2007 (18) | 79 | F | UAP, vomiting | Yes | None | Stomach | Na | Laparotomy |
| Duarte, 2002 (19) | 36 | M | AP, vomiting | No | None | ileum | NA | Laparotomy |
| Gants, 1953 (20) | 30 | M | UAP, vomiting | No | None | Stomach, transverse colon | Abdominal Trauma | Laparotomy |
| Joao, 2022 (21) | 44 | M | UAP, vomiting | Yes | None | Jejunum | Open Appendectomy | Laparotomy |
| Li, 2017 (22) | 38 | F | UAP | No | Gastrocolic lig | ileum | None | Laparotomy |
| Rathnakar, 2016 (23) | 54 | M | AP, vomiting | No | None | Jejunum | None | Laparotomy |
| Tran, 1991 (24) | 24 | F | UAP, vomiting | Yes | None | Jejunum | None | NA |
